# A qualitative study on perceived sexual intimacy and mental health in later life during the COVID-19 pandemic

**DOI:** 10.1192/j.eurpsy.2023.545

**Published:** 2023-07-19

**Authors:** S. von Humboldt, G. Low, I. Leal

**Affiliations:** ^1^William James Center for Research, ISPA - Instituto Universitário, Lisbon, Portugal; ^2^Faculty of Nursing, University of Alberta, Edmonton, AB, Canada

## Abstract

**Introduction:**

The COVID-19 pandemic may affect sexual intimacy and have implications for overall sexual well-being.

**Objectives:**

This study comprised two main objectives: 1) To explore the influence of COVID-19 pandemic on older couples’ sexual intimacy; and 2) To assess how older couples’ sexual intimacy during the COVID-19 pandemic influences mental health.

**Methods:**

The sample of this qualitative study consisted of 391 older participants (between 65 and 87 years of age).

**Results:**

For the first objective, semi-structured interview data yielded five main themes: (1) Less sexual satisfaction (68%); (2) Less sexual desire (67%); (3) Stronger affective relationships (34%); (4) Fear of contracting physical illness (29%); and (5) Less attractiveness (23%). Three main themes concerning mental health were reported by participants: (1) Less anxiety and distress (78%); (2) Greater attention to negative emotional states (55%); and (3) Less emotional outbursts (41%).

**Conclusions:**

The pandemic affected older adults’ sexual intimacy, mostly negatively. Less sexual satisfaction and desire were felt by these older couples. Conversely, stronger affective relationships were reported. In spite of these mostly negative influences, existing sexual intimacy was mostly linked to less perceived anxiety and distress, greater attention to negative emotional states, and less emotional outbursts. Sexual intimacy during COVID-19 has received little attention; however, these results highlight its positive contribution to mental health and therefore a relevant approach to this topic should be taken, especially in later life.

**Disclosure of Interest:**

None Declared

